# Using Frameshift Peptide Arrays for Cancer Neo-Antigens Screening

**DOI:** 10.1038/s41598-018-35673-0

**Published:** 2018-11-26

**Authors:** Jian Zhang, Luhui Shen, Stephen Albert Johnston

**Affiliations:** The Biodesign Institute Center for Innovations in Medicine, Arizona State University, Tempe, AZ 85287 USA

## Abstract

It has been demonstrated that DNA mutations generating neo-antigens are important for an effective immune response to tumors as evident from recent clinical studies of immune checkpoint inhibitors (ICIs). Further, it was shown that frameshift peptides (FSP) generated in tumors from insertions and deletions (INDELs) of microsatellites (MS) in coding region are a very good correlate of positive response to PD1 treatment. However, these types of DNA-sourced FSPs are infrequent in cancer. We hypothesize that tumors may also generate FSPs in transcription errors through INDELs in MS or by exon mis-splicing. Since there are a finite number of predictable sequences of such possible FSPs in the genome, we propose that peptide arrays with all possible FSPs could be used to analyze antibody reactivity to FSPs in patient sera as a FS neo-antigen screen. If this were the case it would facilitate finding common tumor neoantigens for cancer vaccines. Here we test this proposal using an array of 377 predicted FS antigens. The results of screening 9 types of dog cancer sera indicate that cancer samples had significantly higher antibody responses against FSPs than non-cancer samples. Both common reactive FSPs and cancer-type specific immune responses were detected. In addition, the protection of a common reactive FSP was tested in mouse tumor models, comparing to the non-reactive FSPs. The mouse homologs non-reactive FSPs did not offer protection in either the mouse melanoma or breast cancer models while the reactive FSP did in both models. The tumor protection was positively correlated to antibody response to the FSP. These data suggest that FSP arrays could be used for cancer neo-antigen screening.

## Introduction

The research into, and clinical use of, checkpoint inhibitor immunotherapeutics (ICI) has pointed to the importance of neo-epitopes in an effective anti-tumor immune response^1–3^. An even more effective predictor are the frameshift neo-epitopes generated by microsatellite instability (MSI)^4–6^. These peptides are produced when there is a failure to repair INDELs in microsatellites during DNA replication. However, these types of FSP neo-epitopes are infrequent in tumors. Personal cancer vaccines therefore are largely focused on the much more frequent neo-epitopes produced by point mutations in the DNA. For example, of the 254 peptides used in the two clinical trials of personal vaccine reported^7,8^, only 7 were FSPs. Here we explore whether FSPs could be more frequently produced in tumors through RNA processing errors and used as vaccines.

A new and exciting development in treating cancer are personal vaccines. In general, the process involves sequencing the DNA of tumors to find neo-epitopes. These are confirmed to be expressed at the RNA level. Since most neo-epitopes are not immunogenic, an algorithm is used to predict the ones most likely to create an anti-tumor immune response as a vaccine^9^. To ensure at least one reactive component, the vaccines have 10–20 different neoantigens. In most cases, a direct test for the presence of the neo-epitope peptide or an immune response to it is not envisioned before the vaccine is manufactured and administered to the patient. The process is estimated to take 1–3 months.

In the two recent reports of a clinical trial of personal vaccines for late stage melanoma^7,8^ there are several important conclusions. First, two of the total 10 patients were excluded for low number of mutations in one of the trials. Second, even though the algorithm used selected for optimal MHC I-binding peptides, the majority of the immune-reactive peptides elicited MHC II responses. Third, the FS peptides (ADM2, DHX40, RALGAPB) used in the trial elicited strong immune responses. Fourth, the vaccines seemed safe and, though the number of patients were small and screened, the results were encouraging.

It is anticipated that combining these vaccines with checkpoint inhibitors will improve the number of patients responding to the immunotherapeutics. Currently the best indicator of response is a defect in microsatellite repair (MSI+)^4,6^. The first tumor-type agnostic therapeutic (Pembrolizumab) was approved by the FDA based on the strength of this correlation. The limitation is that, except for a few cancers (e.g. colon), the percent of MSI+ patients is low. The high positive response rate of renal cancer to ICI treatment correlates with high number of FS antigens^2^. FS antigens appear to be important drivers of the positive immune response to tumors.

Given the anti-cancer potential of FS neo-antigens we thought to explore whether there were sources in tumors other than those arising from DNA mutations. One obvious source could be INDELs in microsatellites in RNA arising from mis-transcription^10,11^. Since the RNA polymerase does not have an efficient proof-reading function (3′to 5′ exonuclease activity), these errors might be frequent. Another source would be mis-splicing of exons. This process is more error prone in tumor cells^12,13^. The high immunogenicity of the FSPs could induce high immune responses in cancer patients. We reasoned that since the FSP from these errors are predictable from primary sequences we could screen the FS neo-antigens in cancer patient by analysis the antibody response to FSPs with a FSP array.

In light of this background, we are interested in developing cancer vaccines composed of FS antigens. We intend to test both therapeutic and prophylactic vaccines in dogs first, before going human trials. Cancer occurs in dogs at essentially the same frequency as in humans (~30%) and several of their cancers are very similar to that in humans. In addition, the cost of the dog trials are less than for humans. For these reasons, we chose to test the concept of screening FS peptide arrays for cancer vaccine components using dog FSPs.

## Results

### Dog frameshift peptide array platform

Bioinformatic analysis of the dog genome reveals ~7,000 microsatellites of greater than 7 bp homopolyer runs in coding sequences. We chose 322 peptides of the 14,000 possible FS peptides based on the length of the homopolymer and the resulting FS peptide from an INDEL. Also by informatic analysis, there are ~220,000 FS peptides that could result from exon mis-splicing. We chose 19 of these which were discovered in human tumor EST libraries^14^ (high frequencies in tumor EST libraries and low frequencies in normal EST libraries) and were homologs of dog sequences. Another 36 FS antigens were human FS sequences (MS and mis-splicing FSs) that were also discovered from the human EST library analysis and were highly conserved in the dogs^14^. Based on these criteria, 830 peptides from 377 predicted frameshift antigens were synthesized commercially and printed on NSB9 (NSB Postech, Seoul, South Korea) amine surface slides. The space between each peptide on the NSB9 surface is 3 nm and the NSB9 slide is specifically designed for detecting high affinity, cognate antibody-peptide binding, as opposed to the immunosignature arrays which rely on avidity binding^15^. Each frameshift antigen was represented with 1–4, 17-mer non-overlapping frameshift peptides, depending on the frameshift peptide length, with a three-amino acid linker (GSC) at the C-terminus.

**Figure 1:**
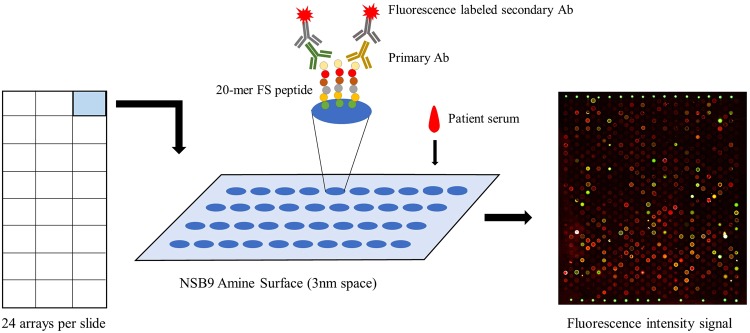
The process of measuring the anti-FS immune response on frameshift peptide arrays. Cancer patient sera was incubated overnight on the frameshift peptide array, fluorescence labeled secondary antibody was then used for transforming antibody-FSP binding signal to fluorescence, each slide contains 24 arrays.

Each NSB9 amine slide included 24 peptide arrays. The principle of this frameshift peptide array was like a conventional ELISA but with much higher sensitivity^16,17^. Cancer patient sera was incubated with the frameshift peptide array overnight and fluorescence labeled secondary antibody was then applied. The slide was scanned afterwards and the anti-frameshift peptide response was transformed to fluorescence intensity (Fig. 1). To test this platform, we screened dog cancer sera samples and non-cancer samples on this array.

### Screening dog cancer serum samples with frameshift peptide arrays

9 types of dog cancer serum with a total of 116 samples were screened on the dog frameshift peptide array. These 9 types of cancer included carcinoma, fibrosarcoma, hemangiosarcoma, lymphoma, mast cell tumor, osteosarcoma, histiocytic sarcoma, synovial cell sarcoma and malignant histiocytosis. 52 age-matched, non-cancer dog sera samples were used as control. The sample description is summarized in Table S1. There were 26 breeds represented.

**Figure 2:**
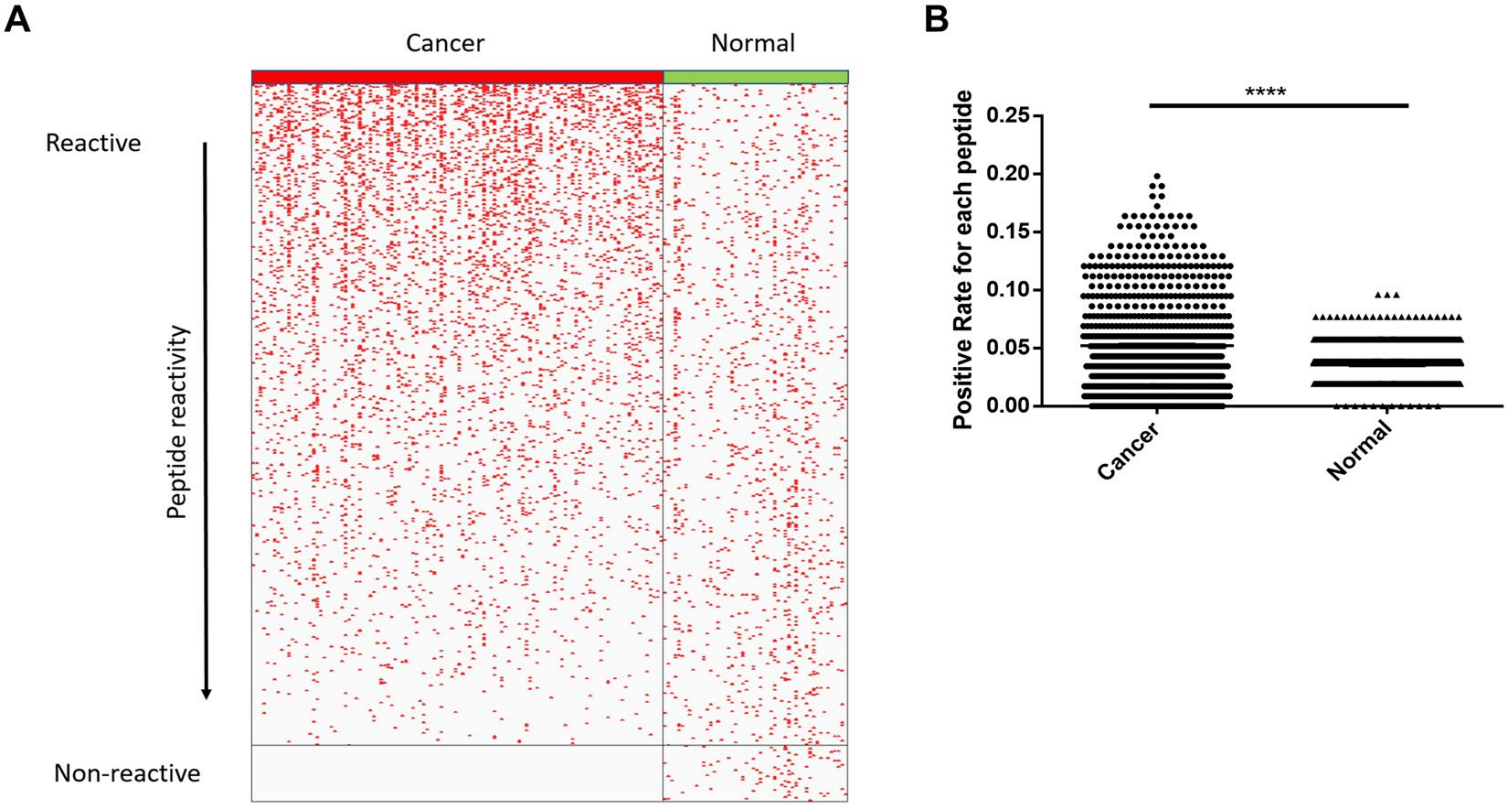
Dog cancer serum samples had higher positive rate than normal samples. (**A**) Overall distribution of FSP positive samples in cancer group and normal group. Reactivity was sorted by descending order of cancer group, each red dot represented one positive sample for corresponding FS peptide. (**B**) Positive rate distribution of each FS peptide was compared between cancer group and normal group of all FSPs, p-value < 0.0001 with two-tail student’s t-test.

The fluorescence intensity for each array (sample) was normalized to the median florescence of the array. A cutoff value (Cutoff = Average (Control) +2*Standard deviation (Control)) was set for each peptide. Samples with normalized values which were larger than the cutoff were counted as positive for this peptide and samples with lower value were negative. The distribution of positive samples for each peptide was shown in Fig. 2. There were fewer positive samples in the non-cancer group and these samples were distributed relatively evenly across all samples. The cancer samples had a significantly higher positive rate than normal samples (p-value < 0.0001, student’s t-test). About half of the peptides had a similar positive rate between the cancer group and non-cancer group, while there was a small group of FSPs (122) which were highly positive in the cancer group with over 10% positive rate. The group with highest positive rate peptides (over 15% positive rate) is listed in Table S2. Most of the listed FSPs were from the MS regions with a positive rate as high as 15%~19%. Interestingly, 65 FSPs had no reactivity in the cancer group at all, while these non-reactive FSPs had the same reactivity in normal group as other FSPs. These differences were highly significant (p-value < 0.01, student’s t-test). There was no obvious difference in reactivity by breed, though the numbers of each breed were too small to detect small differences (Fig. S2). The non-reactive peptides and common reactive peptides were the source of the vaccines tested in the mouse models.

Since 9 types of cancer serum were tested with the array, one interesting question would be whether cancer type-specific and common anti-FS reactivity could be observed.

### Anti-FS reactivity in different cancer types

Out of the total 9 cancer types, 6 cancer types had 10 or more samples in the experiment (Table S1) and these 6 cancer types were used for the cancer type analysis. The positive rate (percentage of positive cancer samples in each cancer type) distribution of all the FSPs was compared between different cancer types.

Frameshift variants in both MS and mis-splicing are depend on the level of RNA production. The FS variants level depends on the RNA expression level of the corresponding genes. So overall, the gene expression is correlated with the abundance of the FSPs, and further correlated with the immune response to these FSPs in cancer patients. Since different cancer types have different gene expression profiles^18^, we would assume that the antibody reactive profiles to FSPs were different in different cancers.

**Figure 3:**
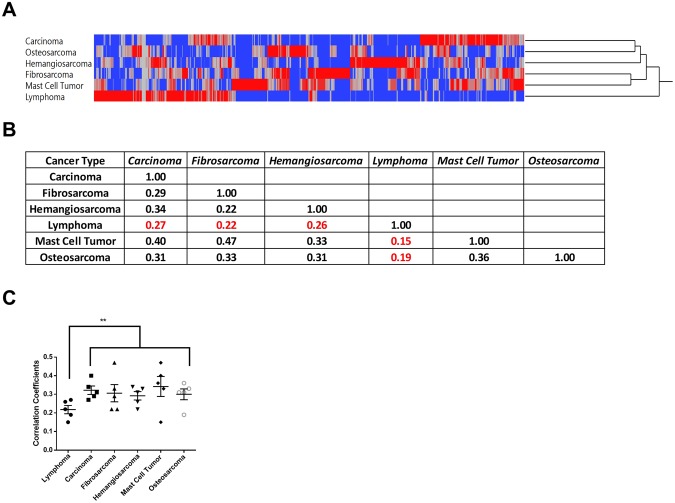
Positive rate distribution across different cancer types. (**A**) Two-way hierarchical clustering of positive rate of each FS peptide by each cancer type. Lymphoma was clustered to a unique group and the rest of the five cancer types as one group. (**B**) Correlation matrix of positive rates by each cancer group (carcinoma). (**C**) Correlation coefficients of each cancer type to the rest 5 cancer types, lymphoma had significant lower correlation coefficients (p-value < 0.01, student’s two tail t-test). Error bar represents standard error (Mean ± SEM).

The positive rate correlations between cancer groups were relatively low (~0.3), which is consistent with our assumptions. However, common reactivity across 3~4 cancer types was common (Fig. 3A), which indicates a broadly effective cancer vaccine would be feasible if we had included enough FSPs to cover different cancer types.

In addition, lymphoma was clustered as a unique group and it had a specific anti-FSP profile which was quite different from the other 5 cancer types. The correlations of positive rate profile between lymphoma and the other cancer types were the lowest, ranging from 0.15~0.26 (Fig. 3B), which further validated our assumptions since lymphoma evolves from a single population of lymphocytes. Another report also demonstrated that lymphoma had a distinct humoral profile compared to other cancer types^19^.

We then tested whether the common reactive FSPs and non-reactive FSPs offered protection in mouse tumor models.

### Only Reactive FSPs provided protection in mouse tumor models

As mentioned previously, there was a group of 65 non-reactive FSPs which had 0% positive rate in the cancer group, while they had the same overall reactivity as observed for all FSPs in the non-cancer group (Fig. 1A). At the same time, there were more than 400 FSPs which had more reactivity in cancer group than non-cancer group. We hypothesized that the reactive peptides in the cancer group could be good vaccine antigens, as most FSPs contain predicted MHC I and II epitopes for humans^2^ and mice (not shown). Two other ideas support this hypothesis: 1. Recent ICI clinical trials showed that ICIs were very effective for late stage microsatellite instability high (MSI+) patients^4,5^ and the FDA granted accelerated approval for using MSI- + as a biomarker for ICI treatments. ICI treatment boosted the anti-MS FSPs immune response for reactive FSPs but not for non-reactive FSPs in these patients, and the boosted anti-reactive FSPs immune response could kill tumor cells effectively; 2. The immunoediting process suppresses the anti-tumor immune response and is constantly removing highly immunogenic tumor cells. The fact that a high anti-FSPs immune response could be detected at late stage may indicate that it was difficult for tumor cells to get rid of these FSPs. However, it was not clear to us what the antigenic potential of the non-reactive FSPs would be.

To test this hypothesis concerning the reactive FSPs and gain information on the non-reactive FSPs, we tested these reactive and non-reactive FSPs in two mouse tumor models. Within these 65 non-reactive FSPs, we found 6 highly conserved mouse homolog peptides, 2 mouse MS FSPs which had the same MS regions at the same location of the homolog genes, 4 mis-splicing FSPs - 2 peptides resulting from mis-splicing events in gene-fusions and 2 from mis-splicing events within the same gene.

**Figure 4:**
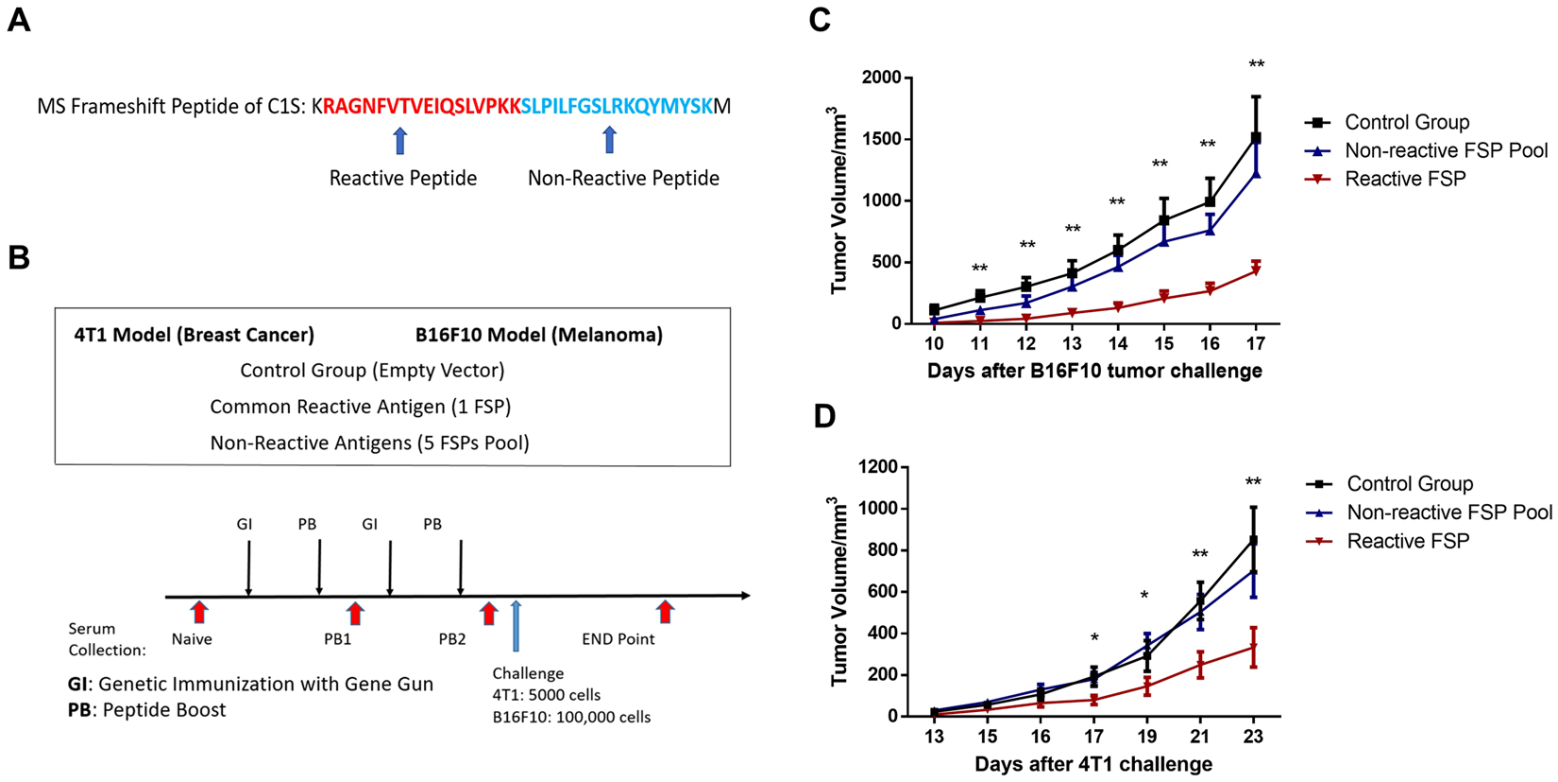
Reactive FSPs showed protection in the mouse melanoma and breast cancer models. (**A**) Reactive and non-reactive FSPs from the same microsatellite frameshift antigen of C1S. (**B**) 3 groups of mice were used: the control group was immunized with control plasmid, non-reactive FSPs pool and reactive FSP. All 3 groups received 2 rounds of genetic immunization with gene gun and peptide boost via subcutaneous injection. The B16F10 cell line was used for the melanoma model and the 4T1 cell line was used for breast cancer model. Each group had 10 mice. (**C**) Reactive FSPs slowed tumor growth significantly compared to the non-reactive FSPs pool and the control group in the melanoma model (student’s t-test, p-value < 0.01), error bar represented mean ± SEM. (**D**) The Reactive FSPs offered tumor protection in mouse breast cancer model as well. The tumor volume was significantly lower than control group and non-reactive FSPs pool group (student’s t-test, p-value < 0.05).

Most frameshift antigens included in the FS array were more than 34 amino acids long so they were represented by 2 or more non-overlapping 17-mer peptides with a GSC linker. The antibody response for different FSP peptides from the same FS antigen usually had different positive rates in the cancer group. To make a direct comparison of reactive peptides and non-reactive peptides, we searched for reactive FSPs from the same 6 frameshift antigens which had one peptide in the non-reactive section. We could find one reactive peptide (RAGNFVTVEIQSLVPKK) from the C1S gene which had 7.7% positive rate in cancer group, the other FSP (SLPILFGSLRKQYMYSK) from the same antigen had no reactivity in cancer group at all (Fig. 4A and Table 1).

**Table 1:** List of non-reactive peptides and reactive peptide.

Candidate	Type	PR in Cancer	Type of FS	Array sequence	Mouse homolog peptide
PCBP2	Non-reactive	0%	Microsatellite	HDAWQHRIQCRFGCICSGSC	HDPWQHRIQCRFGCICS
C1S	Non-reactive	0%	Microsatellite	SLPILFGSLRKQYMYSKGSC	ALPILIGSPIRQNTCLK
FANCI	Non-reactive	0%	Mis-splicing	VSPGVSELRRNSKKYGKGSC	LSPGMSELQRNSKHCGK
CCDC13_HHATL	Non-reactive	0%	Mis-splicing	PLVPAAAAWSLCGPLCGGSC	PMVPAAAAWPLYGPLCG
DDIT3_MARS	Non-reactive	0%	Mis-splicing	LPLGVSRGFPSAKASCFGSC	LPLGVSMGFPSAKANCF
RBM14_RBM4	Non-reactive	0%	Mis-splicing	DVVKGSCQDGEAVHRKPGSC	DVVKGSCQDGEAVHWKS
C1S	Reactive	7.76%	Microsatellite	RAGNFVTVEIQSLVPKKGSC	RVGSFVTMEIPSPVLKK

The mouse melanoma injectable model (B16F10 cell line) and breast cancer injectable model (4T1 cell line) were used for testing the one reactive peptide and a 6 non-reactive peptide pool. A control group received a mock vaccination. All 3 groups received two rounds of genetic immunization with the gene gun on the ear and a peptide boost via sub-cutaneous injection. The reactive peptide vaccine group had significantly slower tumor growth in the melanoma model than the non-reactive FSPs pool group and control group (p-value < 0.01, student’s t-test). The same results were found in the 4T1 breast cancer model - the reactive FSPs offered tumor protection while non-reactive FSPs pool did not. The final tumor volume difference between the non-reactive FSPs pool group and the control group was not significant. These data indicate that reactive FSP is a better cancer neo-antigen in these two different mouse cancer models, and indicate it may be feasible to select cancer FS antigens for multiple cancer types based on the screening results of the frameshift peptide array.

Since these FSPs were first selected based on antibody response against FSPs in dog cancer serum samples, one interesting question would be whether we could see a correlation between the antibody response level against the reactive FSP and tumor protection level.

### Tumor protection is positively correlated to antibody response

Reactive FSPs and non-reactive FSPs were selected based on the antibody response against the FSPs in dog cancer patients. We hypothesized that these antibody responses had anti-tumor activity or it was indirectly related to other anti-tumor mechanisms, for example CD4 activation. To test the idea, the antibody response against vaccinated FSPs was measured in the mouse breast cancer model and compared to tumor volume at the endpoint of the experiment.

**Figure 5:**
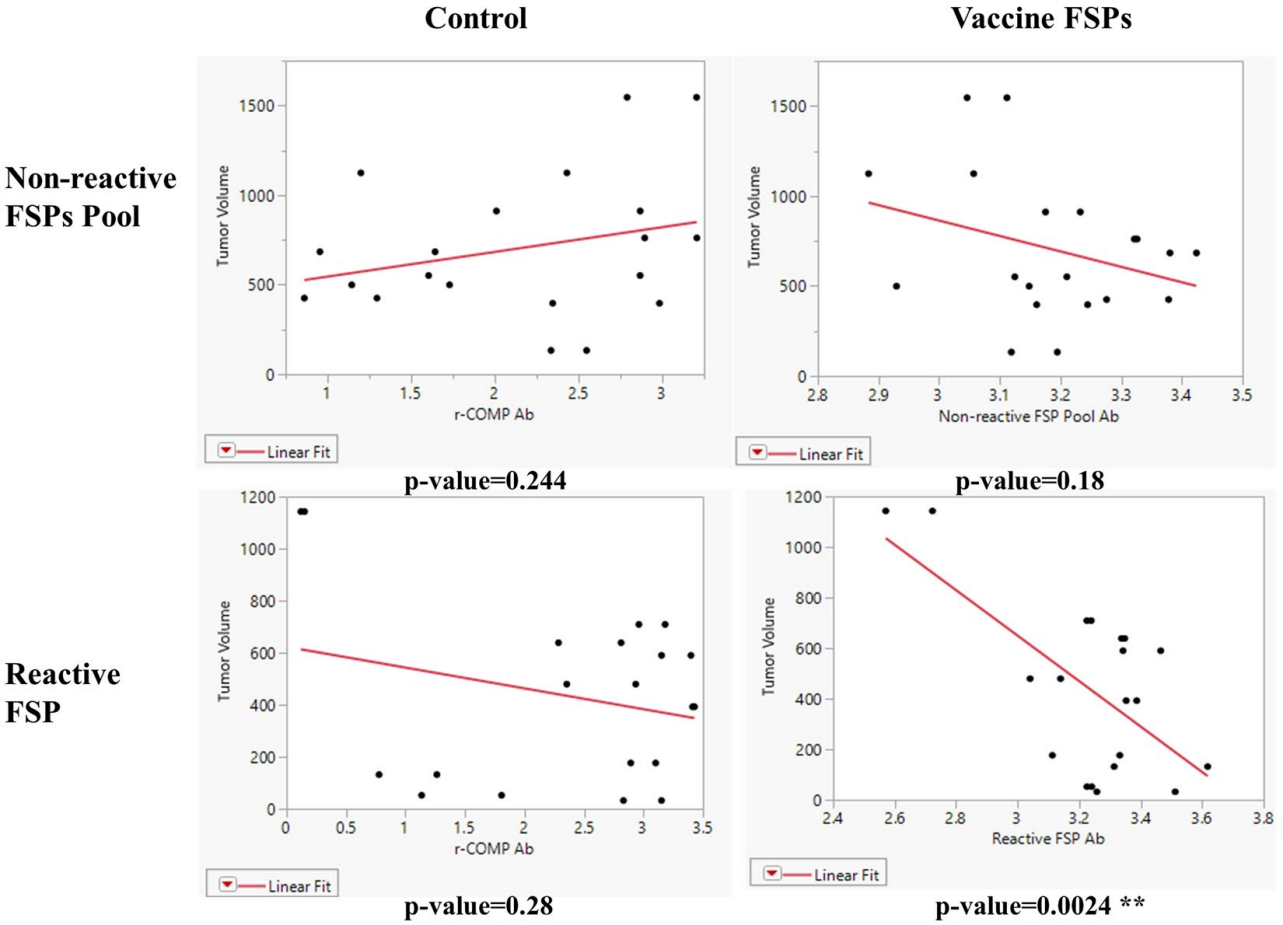
Tumor volume was linearly correlated to antibody response against vaccinated reactive FSPs in the 4T1 breast cancer model. Tumor volume at the end point was used for analysis. The antibody response was measured by ELISA after PB2 and at the end point. R-COMP peptide was encoded in a plamsid and used as an internal control for genetic immunization. The p-value was calculated for linear fit of antibody response against tumor volume.

Antibody responses were measured after PB2 (before tumor challenge) and at end point (after tumor challenge), both time points were included in the analysis since both timepoints could have an impact on tumor protection. The r-COMP peptide is encoded in a plasmid as an internal positive control for genetic immunization. Antibody response against r-COMP was not correlated to tumor volume in the non-reactive FSPs pool group or reactive FSP group, indicating that anti-r-COMP immune response was not essential for tumor protection. However, the antibody response against the reactive FSP was significantly positively correlated to tumor volume at the end point (p = 0.0024 for Reactive FSP, Fig. 5), while antibody response against non-reactive FSPs pool did not correlate to tumor volume (p-value = 0.18, Fig. 5). These data support that tumor protection was positively correlated to antibody response against selected reactive FSPs but not non-reactive FSPs, and the antibody response against reactive FSPs had tumor-killing activity or it was indirectly related to other tumor-killing activity mechanisms.

### Reactive FSP induced a positive T cell immune response and it was related to tumor protection

**Figure 6:**
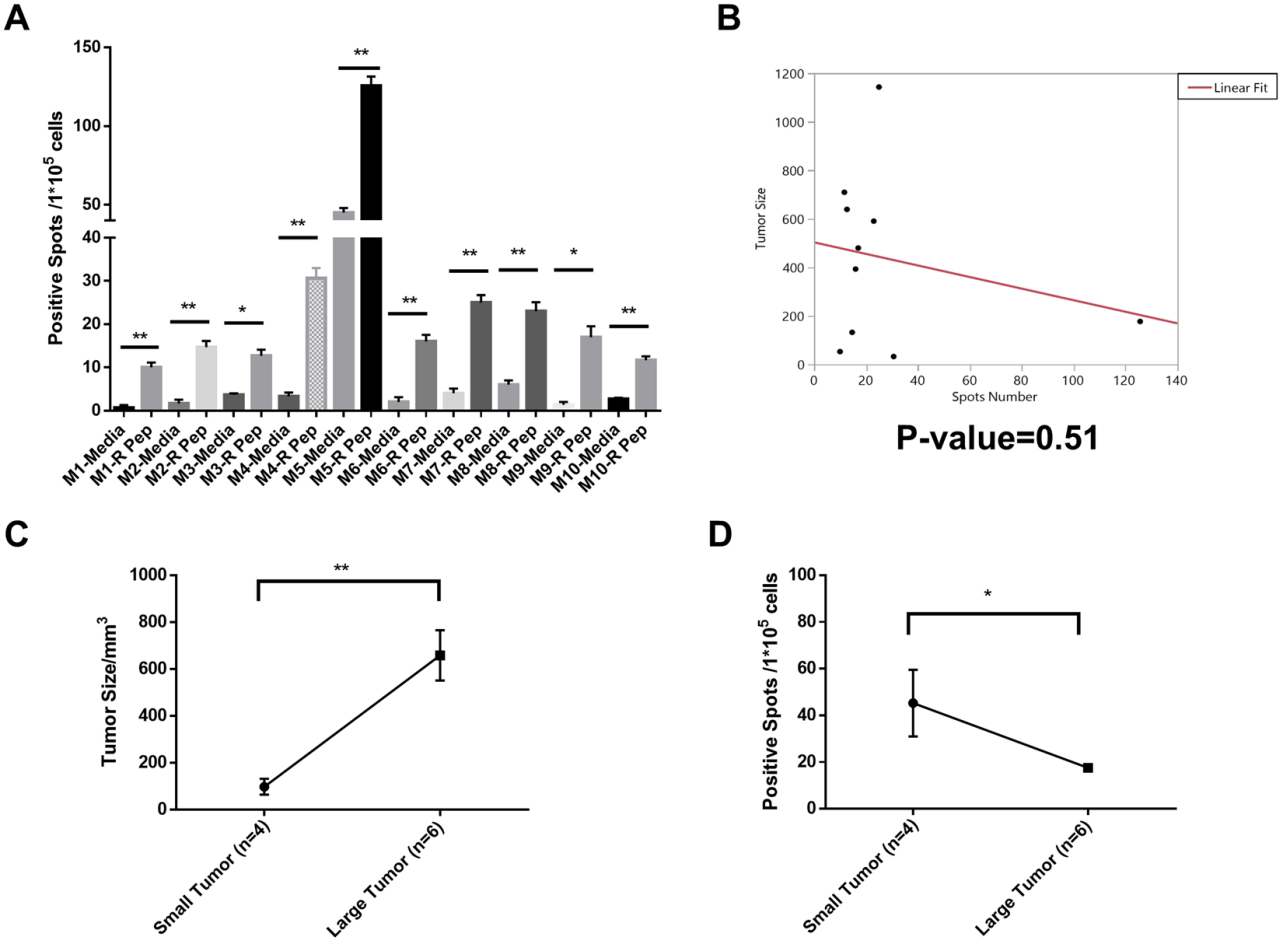
Reactive FSP induced T cell response and it was related to tumor protection in 4T1 breast cancer model. (**A**) IFN-γ Positive spots for media only and reactive FSP (R Pep) in 10 mice (M1-M10) of reactive FSP group. P-value < 0.01 with student’s two-tail t-test. (**B**) Tumor volume was linearly fitted against IFN-γ Positive Spots from ELISPOT experiments, p-value = 0.51. (**C**) 10 mice were separated into Small Tumor group (n = 4) and Large Tumor group (n = 6), p-value < 0.01 with student’s two-tail t-test. (**D**) Positive Spots from Small Tumor group were compared to Large Tumor group, p-value < 0.05 with student’s two-tail t-test. All error bars represent Mean ± SEM.

The T cell immune response is critical in cancer elimination^20^. Here the T-cell immune responses against vaccinated FSPs were measured with IFN-γ releasing enzyme-linked immunospot (ELISPOT) assay. Splenocytes of each mouse were collected at the endpoint. All 10 mice (M1-M10) in the common reactive FSP vaccine group had significantly more positive spots compared to media control (Fig. 6A), which indicated the common reactive FSP induced positive T cell immune response in all the mice.

The ELISPOT spots numbers were linearly fitted against tumor volume in the FSP vaccine group as was done in the ELISA analysis. As evident in Fig. 6B, there was no simple correlation between tumor size and ELISPOT number. However, the single mouse with much higher spot number also had one of the lowest tumor volumes. As another approach to analysis, the 10 mice in the FSP vaccine group were separated to two groups (Fig. 6C): Small Tumor group (n = 4, tumor size < 400 mm^3^) and Large Tumor group (n = 6, tumor size > 400 mm^3^). In this case, the Small Tumor group had significantly more IFN-γ releasing spots than the Large Tumor group, which indicates that T cell immune response was related to tumor protection.

The T cell immune response was also measured against individual FSPs in the non-reactive FSP group. Splenocytes of the mice in this group were pooled for the ELISPOT assay. Results showed that the non-reactive FSPs also induced positive T cell immune responses (Fig. S1).

## Discussion

We first described a new platform for screening potential cancer frameshift antigens that may be produced by RNA mis-processing. 377 frameshift antigens were represented in 830 FSP peptides on the array. We screened 116 dog cancer serum samples representing 9 cancer types and 52 non-cancer serum samples on these arrays. Sera from tumor patients had much higher overall reactivity on the arrays than sera from non-cancer subjects. Cancer-type specific and common anti-FSP immune responses were observed. Lymphoma clustered as a unique group from the other cancer types. We selected non-reactive FSPs and a corresponding reactive FSP based on the array data. We validated that the selected reactive FSP could offer tumor protection in the mouse melanoma and breast cancer models while the non-reactive FSPs did not. We further showed that this tumor protection was linearly correlated to the antibody response against the immunized reactive FSPs. We further showed that there were T-cell responses to both the reactive and non-reactive FSPs, and that the amount of reactivity correlated with protection in the reactive FSP group. All these data suggest that using a frameshift peptide array for cancer FS antigen screening is feasible and efficient.

There are ~220 K potential frameshift antigens at the mRNA level in cancer patients that could be generated from INDELs in coding MS or from mis-splicing of exons, meeting our qualifications for length of MS and FSP. It is assumed that a personal vaccine will consist of 10–20 neo-antigens^7,8^. It has been noted that finding sufficient immune reactive neo-antigens for personal vaccines may be a time-consuming and involved task^9^. Using the frameshift peptide array to screen the B-cell immune response in cancer patients could be an assistance in this regard. A potential criticism of using positive FS antigens discovered from late stage cancer patients is based on the logic that the immune response did not stop the tumor progression in the first place. However, the success of using immune checkpoint inhibitors in late stage MSI- + patients confirms that tumors can be killed by activating late stage anti-FS immune responses. It may also be that the FSP induce an antibody response but not a T-cell response.

The dog cancer samples had overall much more reactivity with the FSP than the non-cancer controls (Fig. 2). However, there was sporadic reactivity in the non-cancer samples. In humans it has been shown that non-cancer subjects have antibody reactivity to MS FS at 2–6%^21^. The dogs in the control group were generally older so it is possible their immune surveillance has eliminated a cancer and the reactivity observed are remnants of that response. Alternatively, the reactivity in non-cancer sera could be high affinity cross-reactivity to a mimotope.

Importantly, we demonstrated that the peptides that were positive on the arrays conferred protection in two mouse models, while peptides that were not reactive did not. Even though the peptides were non-reactive on the arrays, they were just as immunogenic at the antibody level as the reactive peptides as vaccines (Fig. 5). The non-reactive peptides were also immunologically reactive at the T-cell level (Fig. S1). We conclude that the difference in protection was not due to lack of immunogenicity at the B or T-cell level. This result supports the general idea that these FS peptides are a rich source of neo-epitopes and that the FS array can be used to screen for positive components for a personal vaccine. This could potentially greatly simplify choosing components for cancer vaccines. We point out, however, it would be possible for a FSP to not elicit an antibody response but still contain effective T-cell epitopes.

We chose to use a DNA prime/peptide boost protocol for the protection assays. This protocol is known to be very effective in raising an immune response^22,23^, but is not as practical to translate to commercialization as it uses two different platforms. However, one of our intentions is to use the FS platform as a protective, cancer vaccine. In this trial we want to use the best protocol to ensure possible success and then address the format in subsequent efforts.

Our most surprising result was that the protection in mice correlated with the level of antibody binding on the arrays. Antibody responses are generally thought not to play a role in tumor protection. In fact, we did not even find a report of a linear correlation of T-cell responses and protection. The question is why would the level of IgG activity correlate with protection against the tumor. We propose that the reactive peptides were protective because they were selected to be 15–50aa long. At this length the majority will have predicted MHC I and II binding epitopes. However, this does not explain the correlation between the antibody reactive levels and protection. One possibility is that the level of antibody corresponds to the amount of FSP produced and therefore the amount of peptide presented on the tumor cell. Another is that the IgG response presumably is associated with CD4 help. FSPs with good CD4 epitopes may promote higher antibody responses to B-cell epitopes in the same peptide. We note that most of the T-cell response in the personal cancer vaccine trials was to CD4 T-cells. If this antibody correlation is generalizable it would afford a simple method to rank FS cancer antigens for vaccines. Therefore, this observation merits further investigation.

We did not find a linear correlation between tumor protection and T-cell response as we measured it (6B). As noted above, this is not surprising as we could not find a report of such a correlation. It has been reported that some neoantigens confer protection but do not have strong T-cell response^24^ and that neoantigens with strong T cell responses fail to have the protection^25^. However, we did find that the T cell immune response was generally related to tumor protection (Fig. 6C,D). The small tumor group had more IFN γ releasing spots than the large tumor group. The difficulty in delineating a strong correlation between T-cell response and the tumor protection may be a reflection of the complexity and noise in the T-cell assay. This relationship would be more easily studied in the dog or human where more immune cells can be collected.

While not the focus of this study, the group of 65, cancer non-reactive FSPs are an unexpected result. These non-reactive FSPs are not by chance based on statistical analysis (p < 0.001 with student’s t-test, assuming cancer and normal group have a normal distribution with equal variance), and most of these non-reactive FSPs exist in the frameshift antigens which included a reactive FSP counterpart as well (Fig. 4A). That the non-cancer samples showed the same level of antibody reaction as the other FS peptides, indicates that these peptides can elicit an immune response. They also induced a T-cell response (Fig. S1). We do not have a definitive explanation for this puzzling observation but offer the following logic: B-cell (antibody) responses, as we noted, generally depend on CD4 help. It also has become clear that CD4 cells are important in tumor cell protection^26,27^. Possibly, there is a strong selection against presentation of specific MHC II peptides for example through elimination of the HLAs presenting them. Therefore, a set of CD4s would not be activated or provide help to the B-cells. The lack of response to the particular FSP we observed would be a byproduct of suppression of these antigens. This hypothesis would be difficult to test in the dog system as it lacks the required immunological reagents. It will be interesting to determine if the same is true in the human profiles where more reagents are available to test different explanations.

We only assayed for 377 of a possible ~220,000 possible FS antigens from INDELs in MS and mis-splicing. Some cancer samples had a very low level of reaction to the FSPs. One possible explanation is that we did not represent the peptides that the tumor presented. It is feasible to make peptide arrays that represent all possible FS peptides^16,28^. It will be interesting to test these arrays against a wide variety of cancer samples. The prediction is that all tumors will have significant FSP reactivity. With broader surveys of tumors it may be possible to find FSP that are frequently reactive across a type of tumor or even all tumors. These peptides could constitute vaccines that are therapeutic against tumor types or possibly even prophylactic. The array-based antibody screen we describe here would make such screening feasible.

In conclusion, these data demonstrate that the frameshift peptide array platform could be used for cancer neo-antigen screening and reactive FSPs selected from the screening can protect mice from tumor challenge while non-reactive FSPs did not. Besides the use of this platform for broadly effective cancer antigen screening, the platform is also expandable and it could be used widely in discovering cancer subtype specific neo-antigens and personalized frameshift antigens in the future. The array may also have use in diagnosis of cancer.

## Materials and Methods

### Frameshift peptide array

830 frameshift peptides were non-overlapping 17-mer peptides with GSC linker at the C-terminus representing 377 frameshift antigens from mis-splicing events and INDELs in microsatellite regions. All the peptides were synthesized by ChinaPeptides Co. (Shanghai, China). The peptide array was printed as previously described by Applied Microarrays, Inc (Tempe, AZ) on NSB-9 amine slides from NSB Postech (Seoul, South Korea). Serum samples were 1:200 diluted with 3% BSA in PBST and 200 µl diluted serum was incubated with the peptide array at R.T overnight. After washing with PBST for 3 times, the array was incubated with 200 ul 5 nM DyLight-649 anti-dog IgG secondary antibody (Jackson ImmunoResearch, West Grove, PA) for 1 hour at R.T. The slide was washed with PBST 3 times, spin dried and then scanned with the Agilent C Scanner (Agilent Technologies, Santa Clara, CA). Fluorescence intensity was analyzed with GenePix Pro 6.0 (Molecular Devices, San Jose, CA). Each array was normalized to the median. The positive rate was calculated by setting a cutoff as average of normal the samples plus two standard deviation of this average.

### Dog serum samples

116 dog cancer serum samples and 52 non-cancer dog serum samples were collected by Colorado State University and detailed cancer type information is in Tables S4-1.

### Mouse tumor models

Animal experiment procedures and protocols were approved by Arizona State University Institutional Animal Care and Use Committee (IACUC, Protocol #1568R). All animal related experiments were conducted in accordance with IACUC guidelines and regulations. 4T1 and B16F10 cell lines were purchased from ATCC and cultured with ATCC recommended protocols. Naïve C57BL/6 and BALB/c mice were purchased from Jackson Laboratory. 100,000 B16F10 cells were injected into C57BL/6 mice 2 weeks after the final peptide boost, and 5,000 4T1 cells were injected into BALB/c mice 2 weeks after final PB as well. Each group had 10 mice.

### Mouse immunization

Genetic immunization was conducted with the Helios Gene Gun System (Life Science Research, Hercules, CA) using previous published protocols^29–32^. The protocols for making gene vaccine bullets was described elsewhere^33^. The DNA fragments encoding the FS peptides were cloned as a C-terminal fusion into the genetic immunization vectors pCMVi-UB and pCMVi-LSrCOMPTT with the Bgl II and Hind III sites and mixed in a 1:1 ratio as the genetic vaccine plasmids. Three adjuvants were encoded by genetic immunization vectors. LTAB is an abbreviation of immunization with a 1:5 ratio by weight of two plasmids, pCMVi-LTA and pCMVi-LTB, expressing the heat-labile enterotoxins LTA and LTB from Escherichia coli. Vectors pCMVi-UB, pCMVi-LSrCOMPTT, pCMVi-LTA (also called pCMVi-LS-LTA-R192G) and pCMVi-LTB were available from the PSI:Biology-Materials Repository DNASU (dnasu.org) at Arizona State University. The first genetic immunization was at week 7. Each mouse was immunized with two sets of bullets and each set included 1 µg plasmid, 0.25 µg LTA-LTB mix adjuvant and 2.5 µg CpG. 4 weeks later, mice were vaccinated with the second genetic immunization with the same dosage. The control group was immunized with empty plasmid and the same dosage of adjuvants. Peptides were conjugated with KLH using Imject Maleimid-Activated mcKLH Spin Kit (Thermo Fisher Scientific, Waltham, MA) using a standard protocol. Peptide immunization was 2 weeks after the second genetic immunization. In the peptide boost, mice in the vaccine group were immunized subcutaneously with 15 µg KLH-conjugated peptide pool and 15 µg poly I:C (Sigma-Aldrich, St. Louis, MO), and the control group was immunized with 15 µg KLH only and the same dosage of Poly I:C.

### ELISA

Mouse serum was collected after peptide boost and the antibody response was measured by ELISA. Briefly, serum samples were diluted 1:200, 96-well plates (Thermo Fisher Scientific, Waltham, MA) were coated with 50 ul 10 µg/ml peptide overnight at 4 °C, then it was blocked with 3% BSA/PBST for 1 hr at 37 °C. After incubation with sera for 1 hr at 37 °C, wells were probed with HRP Anti-mouse goat IgG for 1 hr at 37 °C, then the plate was developed using TMB substrate solution and terminated with 0.5 N HCl. Plates were assessed using SpectraMax 190 Molecular Devices (Molecular Devices, San Jose, CA) at OD 450 nm.

### ELISPOT

Mouse spleen cells were collected at the termination point. BD Mouse IFN gamma ELISPOT set (BD Bioscience, San Jose, CA) was used and standard protocol of the manual was applied. Splenocytes (10^5^ cells per well) were incubated for 48 hours with the vaccinate frameshift peptides (20 µg/ml) or without peptides (media control). After the assay was completed, the spots were counted with SZ-PT Olympus Microscope (Olympus Corporation, Tokyo, Japan).

### Statistics analysis

The statistical calculation software used was GraphPad Prism 7 (GraphPad Software, San Diego, CA) and JMP Pro (SAS Institute, Cary, NC). The data presentation and the statistical test for each experiment are indicated in the legend of the corresponding figures, as well as the samples size and p values.

## Electronic supplementary material

Dataset 1

Supplementary Information

## Data Availability

All data generated or analyzed during this study are included in published article and supplementary information files.
