# Supplementary material for: Using Frameshift Peptide Arrays for Cancer Neo-Antigens Screening
Source: Sci Rep. 2018 Nov 26;8:17366. doi: 10.1038/s41598-018-35673-0 (PMC6255861; doi:10.1038/s41598-018-35673-0)
Supplement: Supplementary file 2 — Supplementary Information [file 41598_2018_35673_MOESM2_ESM.docx]

# **Using Frameshift Peptide Arrays for Cancer Neo-Antigens Screening**

**Author: Jian Zhang, Luhui Shen and Stephen Albert Johnston***

**Author Affiliations:** **The Biodesign Institute Center for Innovations in Medicine, Arizona State University, Tempe AZ 85287, USA**

**Corresponding Author:** **SAJ.** [**Stephen.johnston@asu.edu**](mailto:Stephen.johnston@asu.edu)**, 480 727 0792**

Table S4-1 Description of Dog tumor samples

Table S4-2 High positive rate peptides in the cancer group

PR: Positive Rate

MS: Microsatellites Frameshift Peptides

Figure S1. Non-reactive FSPs induced positive T cell immune response. Splenocytes of mice in non-reactive group were pooled for assay, 2 out of 6 Non-reactive FSPs had significant more spots (p-value<0.001, student’s two-tail t-test). R-COMP was used as positive control. Error bar represents Mean ± SEM.


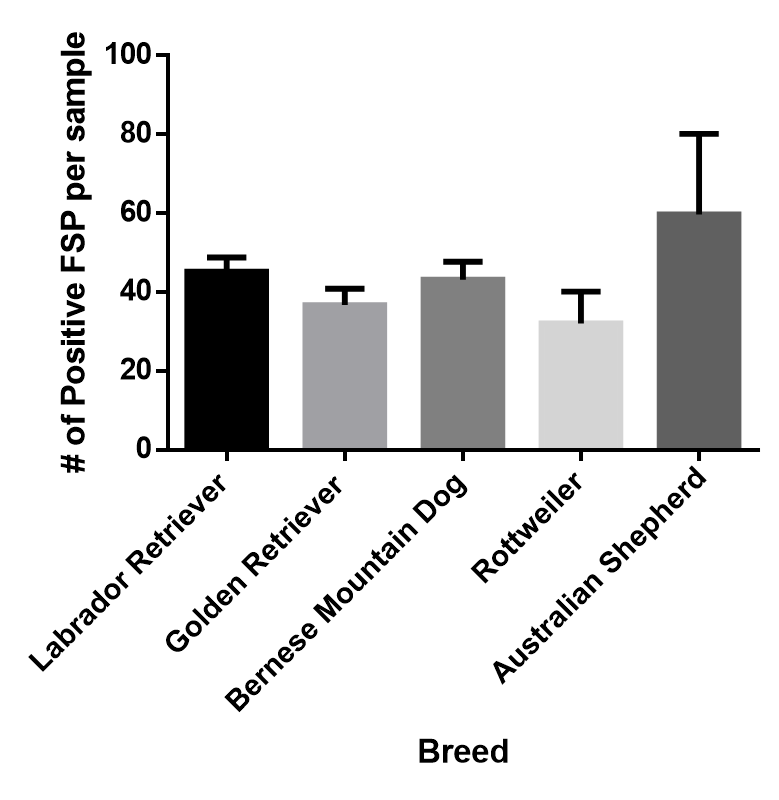


**Figure S2. FSP response across different dog breeds.** Breeds with 3 or more samples were included in the analysis, number of positive FSPs for each sample was used as measurement for overall FS response level. There was no significant difference in every two-group comparison with One-way ANOVA multiple comparison. Error bar represents Mean ± SEM.
